# Assessment of the knowledge, attitude, and perception of the world's population towards monkeypox and its vaccines: A systematic review and descriptive analysis of cross-sectional studies

**DOI:** 10.1016/j.jvacx.2024.100527

**Published:** 2024-08-02

**Authors:** Mohammad Tanashat, Obieda Altobaishat, Abdulrahman Sharaf, Mostafa Hossam El Din Moawad, Mohammad Al-Jafari, Abdulqadir J. Nashwan

**Affiliations:** a Faculty of Medicine, Yarmouk University, Irbid, Jordan; b Faculty of Medicine, Jordan University of Science and Technology, Irbid, Jordan; c Department of Clinical Pharmacy, Salmaniya Medical Complex, Government Hospital, Manama, Bahrain; d Faculty of Pharmacy, Clinical Department, Alexandria University, Alexandria, Egypt; e Faculty of Medicine, Suez Canal University, Ismailia, Egypt; f Faculty of Medicine, Mutah University, Al-Karak, Jordan; g Hamad Medical Corporation, Doha, Qatar

**Keywords:** Monkeypox, Mpo, Vaccine, Knowledge

## Abstract

**Background:**

Prevention and treatment of the monkeypox virus (Mpox) remain challenging in areas where it is endemic. This systematic review and meta-analysis aimed to collect this information from various studies in one study to give a comprehensive view of people's opinions, fears, and behaviors about this virus.

**Methods:**

We searched PubMed, Scopus, Web of Science, the Cochrane Library, and Google Scholar for descriptive cross-sectional study designs conducted in 2022 and 2023 addressing knowledge, attitude, perception, preparedness, willingness to get vaccinated, and practices against Mpox infection.

**Results:**

Among the included studies, 16 studies assessed the level of knowledge of study participants regarding Mpox with a total of 9066 participants. Among them, 4222 (46.6 %) were reported to have good knowledge, and 4844 (53.4%) were reported to have poor knowledge about Mpox. Regarding willingness to get vaccinated against Mpox, 14 studies with a total of 10,696 participants were included. Among them, 7006 (65 %) were willing to get vaccinated while 3690 (35 %) weren’t willing to be vaccinated.

**Conclusion:**

Knowledge about Mpox should be increased and awareness should be spread regarding the importance of preventive measures such as vaccination to protect the population from another COVID-19-like pandemic.

## 1 Introduction

The world was severely affected by the COVID-19 pandemic, and after 3 years of the virus persistence, the monkeypox virus (Mpox) appeared and fear of another pandemic was present worldwide [1]. When the virus was unintentionally discovered in 1958 in monkeys with lesions of a disease that resembled the pox, the illness was given the name monkeypox. The Democratic Republic of the Congo reported the first human case of Mpox in 1970 [2], [3]. Mpox is a zoonotic disease so instances are frequently discovered near tropical rainforests where the virus is carried by animals. Transmission from person to person is scarce. It can spread by coming into contact with bodily fluids, skin lesions, internal mucosal surfaces like the mouth or throat, respiratory droplets, and contaminated objects [4]. Additionally, Mpox infection can spread through raw meat contamination and animal bites or scratches [5].

The public's response to an epidemic is influenced by each person's perceptions of the illness and their ability to change their behavior as conditions change [4], [6]. According to the WHO, the greatest way to prevent the spread of Mpox from person to person is through comprehensive public health surveillance, early diagnosis, and high-quality care from doctors [7].

Prevention and treatment of Mpox remain challenging in areas where it is endemic. To prevent the disease from spreading from person to person, isolation, and immunizations might be utilized. At the moment, three vaccines—replicating (ACAM2000), low replicating (LC16m8), and non-replicating (MVA-BN)—have been approved by the WHO for use against Mpox [8]. Two vaccinations that have been verified by the US Food and Drug Administration to prevent MPOX infection are ACAM2000 and JYNNEOS. However, JYNNEOS is linked to fewer side effects than ACAM2000, which may result in serious adverse events like coronary artery disease [8]. Moreover, tecovirimat, a medication used to treat smallpox, is now approved by the European Medical Association to be used to treat Mpox in both people and animals [9].

Good understanding of the nature of the virus is important to take preventive actions and avoid the process of transmission. This can be done by utilizing environmental surveillance which can serve as an additional means of identifying the spread of pathogens within societies. This suggests that keeping an eye on ecological factors of Mpox can shed light on the virus's possible pathways of transmission as well as the function of public health regulations and stakeholders in Mpox risk control [10]. It's possible that this virus will stay contagious in the environment for extended periods of time [11]. This directs toward good prevention and public health actions against the spread of the virus.

It is anticipated that an additional dangerous pandemic will be cleverly hindered as a lesson from the COVID-19 pandemic and the suffering it has inflicted on healthcare systems around the globe [12]. According to a WHO evaluation, it was challenging to control the spread of Mpox because healthcare workers (HCWs) in particular were not knowledgeable about the illness [13].

Therefore, there must be good awareness and appropriate attitudes and actions toward Mpox among the HCWs and the general population. Although some studies showed the adequacy of awareness and attitudes toward Mpox and vaccination against it, other studies showed the absence of awareness and poor knowledge in addition to non-willingness to take the Mpox vaccine. Therefore, the aim of this systematic review and meta-analysis was to provide an overview of the present knowledge, attitudes, willingness to get vaccinated, level of awareness, worry, and perception of risk among the different populations from studies published in different countries to provide more insights into the world’s reaction to Mpox.

## 2 Methods

### 2.1 Search strategy

Our approach used the Preferred Reporting Items for Systematic Reviews and Meta-Analyses (PRISMA) [14] standards to search the following databases: PubMed, Scopus, Web of Science, the Cochran Library, and Google Scholar from the beginning of 2022 until 2nd February 2023. The following keywords, which best describe the objectives of our systematic review and meta-analysis were used in the search strategy: (knowledge[Title/Abstract]) OR (attitude[Title/Abstract]) OR (perception[Title/Abstract]) OR (preparedness[Title/Abstract]) OR (willing*[Title/Abstract]) OR (practice[Title/Abstract]) OR (fear[Title/Abstract]) OR (Worr*[Title/Abstract]) AND (monkeypox[Title/Abstract]).

### 2.2 Eligibility criteria

At least two authors independently screened all studies in the selection and critical appraisal phases, and any difference was referred to a third author. The eligibility criteria included studies conducted in any language, descriptive cross-sectional study designs conducted in 2022 and 2023, and studies addressing knowledge, attitude, perception, preparedness, willingness to get vaccinated, and practices against Mpox infection. Regarding the previously mentioned outcomes, we included all the studies measuring these outcomes (as named by these studies) through validated questionnaires whether validated through previously published articles or with Cronbach alpha calculations. Different scores were used for each item of them, and an overall score was calculated to determine the knowledge, attitude, perception, preparedness, worries, and practices in addition to their willingness to get vaccinated or not.

Knowledge included questions regarding the Mpox virus, treatment, prevention, signs and symptoms. Attitude and perceptions toward the Mpox virus and the intention toward it were included. Positive or good attitude and negative or bad attitude were considered. Attitudes of the participants toward Mpox is assessed by various scales and questions including feeling the importance of infection prevention, and how to avoid this infection. Also, this includes the thoughts about the danger of the Mpox and the possibility of the occurrence of a pandemic. Moreover, this includes reaction toward virus and its prevention. Worries toward the Mpox were also assessed in some studies. Scales were used in other studies to measure the levels of awareness and perception of risk. Regarding willingness, the participants in the included studies were asked if they are willing to get vaccinated or not.

We excluded (i) narrative reviews, scoping reviews, systematic reviews, conference abstracts, case reports, case series, and any study that didn’t address the main objectives. Rayyan [15] was used to screen the articles using titles and abstracts. We excluded articles which used non-validated questionnaires or scores. Two authors independently reviewed the full text of each potential article. Any conflict or disagreement throughout the systematic review and meta-analysis selection process was settled through consensus.

### 2.3 Data synthesis and extraction

Data extraction was independently performed by three authors using a standardized data extraction sheet designed in Microsoft Excel and was revised by a third author independently. The following information was extracted: study ID, country, type of population, age, male-to-female percentage, education level, percentage of COVID-19, the prevalence of knowledge, attitude, awareness, worries, willingness to receive Mpox vaccination, and perceptions of Mpox risk.

### 2.4 Quality assessment

The quality assessment method was carried out independently by two authors. For cross-sectional studies, we used an adapted version of the Newcastle-Ottawa Scale (NOS-CS) [16]. Each question, with the exception of one in the comparison domain (can get two stars), can get one star. As a result, the maximum score for a study is 9, and the minimum is 0. A score of 7 or more stars indicates a high-quality article, whereas 4–6 or fewer stars indicates moderate quality and 1–3 indicates low quality.

### 2.5 Statistical analysis

Descriptive analysis using frequency and percentage was carried out using SPSS V.26 [17] and Microsoft Excel. We conducted subgroup analysis to determine the difference between populations regarding the outcomes of the systematic review and *meta*-analysis using Open Meta Analyst Software [18].

## 3 Results

Our search strategy resulted in a total of 493 articles which became 289 after the removal of duplicates. By title and abstract screening, 37 articles were included in full-text screening which resulted in 30 eligible articles [19], [20], [21], [22], [23], [24], [25], [26], [27], [28], [29], [30], [31], [32], [33], [34], [35], [36], [37], [38], [39], [40], [41], [42], [43], [44], [45], [46], [47], [48] being included in our systematic review and meta-analysis.

### Baseline characteristics

**Fig. 1:**
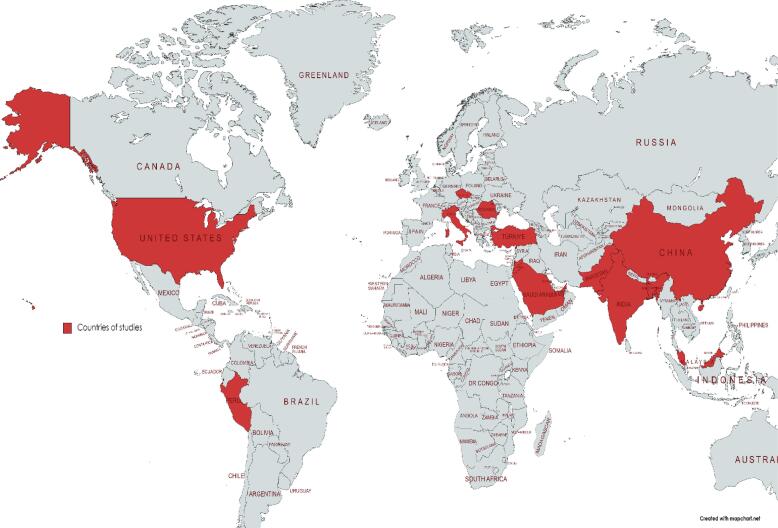
The world map shows the countries where monkeypox assessment studies were carried out.

All the included studies were cross-sectional studies to assess different levels of knowledge, attitude, perception, worries, willingness, and awareness regarding Mpox. Among the included studies, 7 studies were conducted in Saudi Arabia, 4 studies in China, 3 studies in Jordan, 2 studies in Italy, India, Peru, and the USA each, and one study in other countries as shown in Table 1 **and** Fig. 1. The population of the study varied among the included studies as 14 studies included healthcare workers as their main target population, 10 studies were reported to include participants from the general population, 4 studies included medical students and 2 studies included university students in different specialties. Among a total of 5363 participants, 4810 (89.6 %) were reported to be vaccinated against COVID-19. Level of education was reported among 18,093 participants, 10,908 (60 %) were undergraduates and 7185 (40 %) were postgraduates. The age ranged from 19.9 to 54.5 years with 8276 males and 11,704 females of the study participants as shown in the Table 1.

**Table 1:** Baseline characteristics of the study participants.

Study ID	Country	Study design	Population	Age, mean (SD)	Gender (m/f)	Education level		Covid vaccine	
						Undergraduate	Post-graduate	Number	Total
Sallam et al, 2022 (a)	Jordan	Cross sectional	Medical students	19.9 (1.4)	183/432	615	−		−
Zamora et al, 2022	Peru	Cross sectional	Healthcare workers	36.6 (10.3)	269/194	−	463		−
Alshahrani et al, 2022 (a)	Saudi Arabia	Cross sectional	Medical students	−	131/183	314	−	285	314
Lin et al, 2022	Malaysia	Cross sectional	Medical students	−	75/154	229	−		
Kumar et al, 2022	Pakistan	Cross sectional	University students	22.5 (3.5)	432/514	867	79	870	946
Temsah et al, 2022 (a)	Saudi Arabia	Cross sectional	General population, Healthcare workers		650/896422/708	135,680	1,901,047		
Riad et al, 2022	Czech Republic	Cross sectional	Healthcare workers	46.1 (12.0)	35/306		341	311	341
Peng et al, 2023	China	Cross sectional	Health care workers	37.9 (9.4)	208/431	419	220	−	−
Wang et al, 2023	China	Cross sectional	General population	−	798/1337	1419	716	−	−
Meo et al, 2022	Saudi Arabia	Cross sectional	General population	−	466/554	367	652	−	−
Ahmed et al, 2022	India	Cross sectional	Medical students	−	−	302	38	−	−
Ricco et al, 2022	Italy	Cross sectional	Healthcare workers	42.9 (10)	57/106	−	163	163	163
Rodríguez et al, 2022	Peru	Cross sectional	General population	28.31 (9.72)	176/275	310	141	445	451
Gallè et al, 2023	Italy	Cross sectional	General population	54.5 (13.4)	709/643	599	753	1324	1352
Winters et al, 2022	US	Cross sectional	General population	−	410/436	738	118	581	836
Ghazy et al, 2022	Nigeria	Cross sectional	Healthcare workers	median, IQR = 37, 28–48	211/169	288	101	−	−
Temsah et al, 2022 (b)	Saudi Arabia	Cross sectional	General population	−	650/896	353	1193	−	−
Sallam et al, 2022 (b)	Jordan	Cross sectional	Healthcare workers	−	238/368	450	156	−	−
Alshahrani et al, 2022 (b)	Saudi Arabia	Cross sectional	General population	−	198/282	66	414	357	480
Jairoun et al, 2022	UAE	Cross sectional	University students	31.13 (6)	208/350	558	0	−	−
Sallam et al, 2022 (c)	Jordan	Cross sectional	General population	−	178/433	528	83	−	−
Ajman et al, 2022	Saudi Arabia	Cross sectional	Healthcare workers	−	422/708	−		−	−
Hasan et al 2023	Bangladesh	Cross-sectional	Healthcare workers	−	184/205	325	64	−	−
Sahin et al 2023	Turkey	Cross-sectional	Healthcare workers	−	117/166		283	283	283
Alshahrani et al 2022, et al (c)	Saudi Arabia	Cross-sectional	Healthcare workers	−	226/172		398	−	−
Dong et al, 2022	China	Cross-sectional	General population	30	264/257	480	41	−	−
Bates et al, 2022	USA	Cross-sectional	Healthcare workers	−	113/69		197	191	197
Peptan et al, 2022	Romania	Cross-sectional	General population	−	398/412	571	239	−	−
Hong et al, 2022	China	Cross-sectional	Healthcare workers	−	266/766	875	157	−	−
Kaur et al, 2022	India	Cross-sectional	Healthcare workers	−	232/178	235	175	−	−

### Quality assessment

Of the 30 included studies, 16 [19], [20], [21], [22], [23], [24], [25], [26], [27], [28], [29], [30], [31], [32], [33], [34] were of high quality, 12 [35], [36], [37], [38], [39], [40], [41], [42], [43], [44], [45], [46] were of moderate quality and 2 [47], [48] were of low quality **(**Table 2**)**.

**Table 2:** Quality assessment of included studies using New Castle Ottawa Scale.

Study name	Representativeness of the cases(★)	Sample size(★)	Non-Response rate(★)	Ascertainment of the screening/surveillance tool (max★★)	The potential confounders were investigated by subgroup analysis or multivariable analysis(★)	Assessment of the outcome (max★★)	Statistical test (★)	Quality level
Sallam et al, 2022 (a)	★	★	★	★	★	★★	★	High (8)
Zamora et al, 2022	★	★	★	★★	★	★	★	High (8)
Alshahrani et al, 2022 (a)	★	−	−	★	★	★★	★	Moderate (6)
Lin et al, 2022	★	−	−	★★	−	★★	★	Moderate (6)
Kumar et al, 2022	★	★	−	★	★	★★	★	High (7)
Temsah et al, 2022 (a)	★	★	★	★★	★	★★	★	High (9)
Riad et al, 2022	★	−	★	★★	★	★★	★	High (8)
Peng et al, 2023	★	★	−	−	★	★★	★	Moderate (5)
Wang et al, 2023	−	★	★	★	★	★★	★	High (7)
Meo et al, 2022	−	★	−	−	★	★★	★	Moderate (5)
Ahmed et al, 2022	−	−	−	★	−	★★	−	Low (3)
Ricco et al, 2022	★	−	−	★	★	★★	★	Moderate (6)
Rodríguez et al, 2022	−	★	−	★	★	★★	★	Moderate (6)
Gallè et al, 2023	★	★	−	★	★	★★	★	High (7)
Winters et al, 2022	★	★	−	★	★	★★	★	High (7)
Ghazy et al, 2022	★	★	★	★★	★	★★	★	High (9)
Temsah et al, 2022 (b)	−	−	★	−	★	★★	★	Moderate (5)
Sallam et al, 2022 (b)	−	★	★	★	★	★★	★	High (7)
Alshahrani et al, 2022 (b)	−	★	★	★★	−	★★	★	High (7)
Jairoun et al, 2022	−	−	−	−	★	★★	★	Moderate (4)
Sallam et al, 2022 (c)	★	★	★	★	★	★★	★	High (8)
Ajman et al, 2022	−	★	−	★	★	★★	★	Moderate (6)
Hasan et al 2023	★	−	★	−	★	★★	★	Moderate (6)
Sahin et al 2023	★	★	★	★★	★	★★	★	High (9)
Alshahrani et al 2022, et al (c)	★	★	−	★★	★	★★	★	High (8)
Dong et al, 2022	★	★	★	−	★	★★	★	High (7)
Bates et al, 2022	−	★	★	★	★	★★	★	High (7)
Peptan et al, 2022	−	−	★	−	−	★★	−	Low (3)
Hong et al, 2022	−	★	★	−	★	★★	★	Moderate (6)
Kaur et al, 2022	−	−	★	★★	−	★★	★	Moderate (6)

### Statistical analysis

**Fig. 2:**
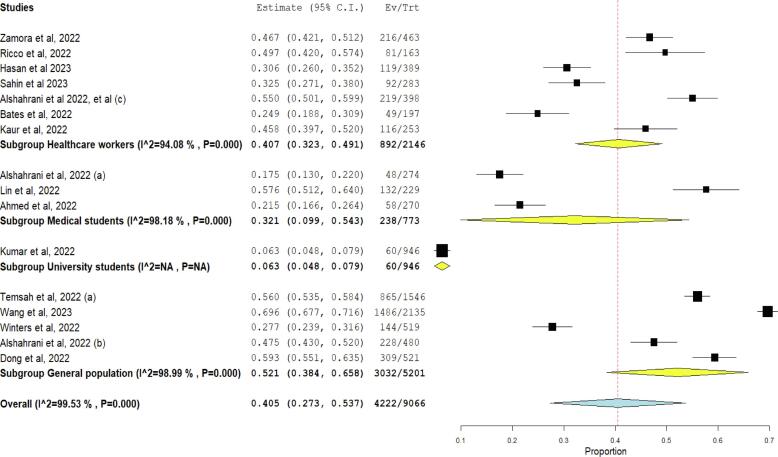
Forest plot of subgroup analysis regarding knowledge of participants about Monkeypox.

Among the included studies, 16 studies assessed the level of knowledge of study participants regarding Mpox with a total of 9066 participants. Among them, 4222 (46.6 %) were reported to have good knowledge, and 4844 (53.4 %) were reported to have poor knowledge about Mpox. By subgroup analysis, the systematic review and *meta*-analysis showed that the knowledge was highest in the general population (58.3 %), followed by HCWs (41.6 %), and then medical students (30.7 %) **(**Fig. 2**)**.

**Fig. 3:**
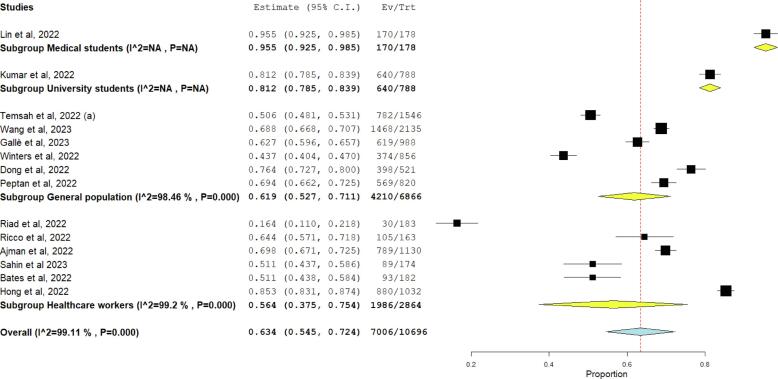
Forest plot of subgroup analysis regarding willingness of participants to get vaccinated against Monkeypox.

Four studies assessed the attitude towards Mpox with a total of 1199 participants, 862 (71.9 %) of them had a good attitude while 337 (28.1 %) had a bad attitude. Regarding willingness to get vaccinated against Mpox, 14 studies with a total of 10,696 participants were included. Among them, 7006 (65 %) were willing to get vaccinated while 3690 (35 %) weren’t willing to be vaccinated (Table 3). By subgroup analysis, HCWS were the most willing population to be vaccinated against Mpox (65.5 %) followed by general population (61.3 %) **(**Fig. 3**).**

**Table 3:** Assessment of level of knowledge, attitude, willingness to get vaccinate, worry, awareness and perception of risk of monkeypox virus.

**Level of Knowledge**		
**Study ID**	**Good**	**Poor**
Zamora et al, 2022	216	247
Alshahrani et al, 2022 (a)	48	226
Lin et al, 2022	132	97
Kumar et al, 2022	60	886
Temsah et al, 2022 (a)	865	681
Wang et al, 2023	1486	649
Ahmed et al, 2022	58	212
Ricco et al, 2022	81	82
Winters et al, 2022	144	375
Alshahrani et al, 2022 (b)	228	252
Hasan et al 2023	119	270
Sahin et al 2023	92	191
Alshahrani et al 2022, et al (c)	219	179
Dong et al, 2022	309	212
Bates et al, 2022	49	148
Kaur et al, 2022	116	137

**Attitude**		
	**Good**	**Bad**
Lin et al, 2022	220	9
Kumar et al, 2022	194	104
Hasan et al 2023	330	59
Sahin et al 2023	118	165

**Vaccine**		
	**Willingness to get vaccinated**	**No willingness**
Lin et al, 2022	170	8
Kumar et al, 2022	640	148
Temsah et al, 2022 (a)	782	764
Riad et al, 2022	30	153
Wang et al, 2023	1468	667
Ricco et al, 2022	105	58
Gallè et al, 2023	619	369
Winters et al, 2022	374	482
Ajman et al, 2022	789	341
Sahin et al 2023	89	85
Dong et al, 2022	398	123
Bates et al, 2022	93	89
Peptan et al, 2022	569	251
Hong et al, 2022	880	152

**Worry**		
	**Number**	**Total**
Temsah et al, 2022 (a)	1156	2676
Peng et al, 2023	362	639
Wang et al, 2023	1127	2135
Meo et al, 2022	412	1020
Ahmed et al, 2022	35	340
Sahin et al 2023	104	283
Hong et al, 2022	277	1032

**Awareness**		
	**Number**	**Total**
Zamora et al, 2022	458	463
Temsah et al, 2022 (a)	676	1130
Peng et al, 2023	459	639
Wang et al, 2023	1164	2135
Meo et al, 2022	799	1020

**T**		
	**Number**	**Total**
Zamora et al, 2022	445	463
Alshahrani et al, 2022 (a)	246	314
Kumar et al, 2022	576	946
Temsah et al, 2022 (a)	1208	2676
Wang et al, 2023	304	2135
Meo et al, 2022	278	1020
Ahmed et al, 2022	102	340
Ricco et al, 2022	80	163

Seven studies with a total of 8125 assessed the worries regarding Mpox, and 3473 (42.7 %) were reported to be worried about Mpox. The level of awareness was assessed in five studies with a total of 5387 participants, 3556 (66 %) of them were reported to be aware of Mpox. Perception of risk towards Mpox was assessed in 8 studies with a total of 8057 participants, 3239 (40 %) of them were reported to have a perception of risk towards Mpox **(**Table 3**)**.

We reported the aims of the studies and the summary of the main findings in a Table 4.

**Table 4:** Summary of the aims and main findings of the included studies.

Study ID	Country	Population	Aim of study	Main findings
Sallam et al, 2022 (a)	Jordan	Medical students	Evaluate students in various health faculties' knowledge of monkeypox, conspiracy beliefs about emerging virus illnesses, and their associated determinants.	– Only 26.2% of respondents were aware that a vaccine to prevent monkeypox is available. – Older participants (>21 years) were significantly related to better monkeypox knowledge. – Older age, female gender, and association with non-medical schools or faculties were linked to higher levels of conspiracy theories about new virus infections. – The findings also show that lesser levels of monkeypox knowledge are related to higher levels of conspiracy theories.
Zamora et al, 2022	Peru	Healthcare workers	Evaluate the amount of knowledge about monkeypox among Peruvian physicians and identify the factors associated with greater knowledge.	– Only 60.7 % of physicians were aware of the monkeypox vaccine. – Only 49.0 % of those who took part were aware of monkeypox proctitis.
Alshahrani et al, 2022 (a)	Saudi Arabia	Medical students	Evaluate medical students' knowledge and attitudes toward the monkeypox virus in Saudi Arabia.	– The majority of participants (93.8 %) believe that the monkeypox virus has spread globally and is more prevalent in homosexual partnerships (87.3 %). – More than half of respondents (56.3 %) stated that direct touch is the most common mechanism of transmission. – Only 41.5 % of respondents agreed that avoiding contact with an infected individual is an important factor in limiting the spread of monkeypox.
Lin et al, 2022	Malaysia	Medical students	Assess preclinical and clinical dentistry students' awareness, knowledge, and attitude toward monkeypox virus infection in Malaysia	– Preclinical and clinical dental students were aware of the presence of monkeypox (89.5 % and 94.4 %, respectively), that the disease had surfaced in non-endemic countries (81.0 % and 87.1 %, respectively), and that the World Health Organization had declared it a public health emergency of international concern (73.3 % and 79.0 %, respectively). – The overall knowledge level of clinical dentistry students was considerably greater than that of preclinical dental students (P = 0.014). – There was no significant difference (P = 0.736) between preclinical (95.2 %) and clinical (96.8 %) dentistry students in their attitudes about monkeypox.
Kumar et al, 2022	Pakistan	University students	Study vaccination knowledge, attitude, views, and willingness among Pakistani university students.	– Most respondents (68.3 %) were unaware of monkeypox before 2022. – In terms of overall understanding of monkeypox, the majority of respondents (76.7 %) had average knowledge, with only a minority having high knowledge (6.3 %). – In terms of overall attitudes towards monkeypox, the majority of responders (68.5 %) were neutral. – The population's willingness to be vaccinated was 67.7 %.
Temsah et al, 2022 (a)	Saudi Arabia	Healthcare workers	Assess the Saudi public's and Healthcare workers understanding of monkeypox, as well as their information-seeking behavior.	– 61.3 % of the general population and 74.2 % of healthcare personnel expressed a desire to learn more about monkeypox; both groups had average overall mean monkeypox knowledge scores. – Members of the public with university degrees and those who expressed high levels of concern about monkeypox had considerably higher knowledge scores. – Healthcare staff had low vaccine knowledge, with only 57 % recognizing that monkeypox can appear in the early stages similar to COVID-19. – Female healthcare workers and those with a strong self-reported awareness of monkeypox had significantly higher knowledge ratings.
Riad et al, 2022	Czech Republic	Healthcare workers	Assess Czech healthcare workers' knowledge of monkeypox and their attitudes toward the monkeypox vaccine.	– Only 8.8 % of individuals accepted to be vaccinated against monkeypox; 44.9 % refused, and 46.3 % were unsure. – The participants exhibited inadequate levels of factual knowledge, particularly about monkeypox vaccines and treatments.
Peng et al, 2023	China	Health care workers	Examine medical workers' perspectives, worries about monkeypox, attitudes about monkeypox vaccination, and their correlations.	– Approximately 71.8 % of people expressed monkeypox perceptions, 56.7 % were worried about monkeypox, and 64.9 % supported the promotion of monkeypox immunization. – Medical workers over the age of 50 who worked in the infectious diseases, dermatology, and venereal diseases departments and correctly answered the monkeypox transmission route was more aware of the monkeypox virus before to investigation. – 30.7 % said they were more concerned about monkeypox than the coronavirus (COVID-19).
Wang et al, 2023	China	General population	Understand the public's views, attitudes perceived preventive measures and vaccination acceptability to monkeypox in China.	– 33.2 % of the participants were more worried about monkeypox than COVID-19. – The majority of participants were willing to take precautions (76.3 % practiced cleanliness, 68.2 % avoided social situations, and 85.9 % avoided travel). – Individuals who were anxious about monkeypox were more willing to take precautions. – 68.8 % of the respondent were willing to use the monkeypox vaccine. – Participants who were older and had a greater income were less likely to use the monkeypox vaccine. – Being more concerned about monkeypox than COVID-19 and having more information about monkeypox were related to a greater desire to consider vaccination.
Meo et al, 2022	Saudi Arabia	General population	Assess the public's perceptions, knowledge, and attitudes toward monkeypox in Saudi Arabia.	– 78.3 % of respondents thought monkeypox disease had become a pandemic, and 78.2 % thought the disease was most frequent in Western and Central Africa. – 62.8 % of participants indicated that health officials should conduct public preventive measures, while 3.7 % suggested that health officials launch monkeypox vaccine campaigns.
Ahmed et al, 2022	India	Medical students	Assess medical students and nursing staff's knowledge and concerns regarding the ongoing Monkeypox outbreak.	– The majority of participants failed to recognize respiratory droplets as a mechanism of transmission; 45 % failed to recognize lymphadenopathy as a clinical characteristic of the disease. and 80.5 % saw contact quarantine as an infection prevention and control measure.
Ricco et al, 2022	Italy	Healthcare workers	Examine a sample of Italian medical professionals' knowledge, attitudes, and practices about monkeypox.	– Around 27.0 % of participants reportedly knew of monkeypox even before the inception of the current outbreak – 78.5 % of the participants acknowledged the potential transmission through the respiratory system, and 74.8 % reported that standard preventive measures may be sufficient to avoid infection. – 60.1 % of the respondents knew that an effective vaccine is available
Rodríguez et al, 2022	Peru	General population	To develop a scale that evaluates monkeypox fear.	– The study makes a psychometrically measure to evaluate symptoms of fear during the monkeypox emergency.
Gallè et al, 2023	Italy	General population	To assess the level of public knowledge about monkeypox.	– Mass media were found to be associated with lower knowledge. – A low level of worry about the transmission of Monkeypox infection was found.
Winters et al, 2022	US	General population	To survey the general population about their Monkeypox vaccination intentions, their knowledge, and trusted sources of information.	– Women are less eager than men to get a Monkeypox vaccine when it is recommended. – Around half of the participants intended to get a Monkeypox vaccine if this is recommended. – Risk perception had a strong positive relationship with vaccination intentions.
Ghazy et al, 2022	Nigeria	Healthcare workers	To assess the psychological antecedents of Monkeypox vaccination among healthcare workers.	– Healthcare workers' psychological antecedents for Monkeypox vaccination pointed to an unsatisfactory attitude against the vaccine. – Complacency is a psychological determinant of Monkeypox vaccination, people who are complacent think that vaccination is unneeded because their immune system can defend them.
Temsah et al, 2022 (b)	Saudi Arabia	General population	To assess the general population’s worries, perception, and vaccine acceptance for COVID-19 and Monkeypox during the first month of the WHO announcement.	– Worry levels among the general population were higher towards COVID-19 than Monkeypox during the first month of the WHO announcement. – Perception of Monkeypox as a dangerous disease, worry about spreading the disease, and high commitment to infection precautionary measures were predictors of acceptance of Monkeypox vaccination. – Old age and high education level are predictors of low acceptance of vaccination.
Sallam et al, 2022 (b)	Jordan	Healthcare workers	To assess their knowledge and their confidence in the diagnosis and management of the disease, also the assessment of their attitude towards emerging virus infections from a conspiracy point of view.	– Low level of Monkeypox knowledge was found among healthcare workers. – More than half of the study participants agreed to some extent that male homosexuals had a role in the spread of human monkeypox.
Alshahrani et al, 2022 (b)	Saudi Arabia	General population	To assess the level of knowledge regarding monkeypox and provide standard information.	– The results showed that more than half of the participants had low knowledge about monkeypox. – Participants with higher education levels, employed, healthcare workers, and high-income earners had higher knowledge of Monkeypox.
Jairoun et al, 2022	UAE	University students	To evaluate the knowledge and awareness regarding disease prevention and treatment. To assess the level of knowledge about Monkeypox source, signs/symptoms, transmission, prevention, and treatment among university students.	– The knowledge of Monkeypox among university students is relatively low. – Better knowledge of human monkeypox was noticed among female participants and participants from medical colleges.
Sallam et al, 2022 (c)	Jordan	General population	To assess the aspect of the general population towards the role of male homosexuals in the Monkeypox spread worldwide and its links to the conspiracy beliefs.	– A majority of participants held conspiratorial beliefs against emerging virus infections. – The agreement to the role of male homosexuals in the Monkeypox spread was associated with older age and higher Monkeypox knowledge.
Ajman et al, 2022	Saudi Arabia	Healthcare workers	To assess the worries and concerns among Healthcare workers, and their acceptance of the Monkeypox vaccine.	– Male HCWs were less worried about Monkeypox than females. – Medical students were significantly more worried compared to the other participants. – HCWs who previously developed COVID-19 were significantly less worried about the Monkeypox outbreak.
Hasan et al 2023	Bangladesh	Healthcare workers	Assessing the readiness of Bangladeshi physicians by assessing their knowledge and attitudes to monkeypox.	– Bangladeshi doctors lack knowledge about monkeypox
Sahin et al 2023	Turkey	Healthcare workers	Evaluate doctor information, mindset issue, and vaccine recognition for monkeypox	– They determined that male HCWs have been appreciably extra informed approximately monkeypox than female – Good information approximately monkeypox improved with age
Alshahrani et al 2022, et al (c)	Saudi Arabia	Healthcare workers	Assess the information and mindset in the direction of monkeypox contamination amongst doctors, frontline healthcare workers	– Negative information is encouraged through a growing age (worse in physicians while getting older than 36 years), gender (worse in males), degree of paintings (experts and consultants), paintings sector (worse in governmental areas), and former scientific training (worse in the ones which have now no longer acquired facts on monkeypox for the duration of scientific faculty or residency years); additionally, there have been no giant variations in step with the specialty, years of experience, vicinity of the country, if one had heard earlier than approximately the disease, and if it changed into the primary time they heard approximately it.
Dong et al, 2022	China	General population	This observation changed into carried out to atone for the lack of expertise and pick out a vaccination purpose evaluation for monkeypox in China	Having a better training degree and being a healthcare employee have been associated with having better monkeypox.
Bates et al, 2022	USA	Healthcare workers	This observation turned carried out to make amends for the lack of information and discover a vaccination goal evaluation for monkeypox	Monkeypox clinicians in Ohio have bad stages of understanding associated with monkeypox and are insufficiently possibly to practice vaccination behaviors to save you from monkeypox.
Peptan et al, 2022	Romania	General population	A study of the vaccination of the Romanian population against the virus that causes monkeypox, with the aim of determining the level of compliance regarding the decision related to vaccination acceptance/non-acceptance/hesitation,	Even though 26.3 % of respondents feared the new disease, they were reluctant to respond to monkeypox, and only 29.3 % agreed to be vaccinated against this spreading disease.
Hong et al, 2022	China	Healthcare workers	This study investigated Chinese health professionals' willingness to receive the monkeypox vaccine and analyzed the factors that influenced their decision.	The study found that most demographics, such as gender, location, education level, occupation, and department, had no effect on immunization, which is inconsistent with previous studies. Multivariate logistic According to regression analysis, the understanding that MPX vaccination is required to control the spread of MPXV was an important factor influencing respondents’ decision to vaccinate
Kaur et al, 2022	India	Healthcare workers	This study was conducted to assess dentists’ knowledge and awareness of monkeypox.	The main finding of this study was the generally inadequate knowledge among study subjects.

## 4 Discussion

Knowledge of the disease, attitudes toward prevention, and intentions to follow advised practices are major determinants of the adoption of preventive measures, especially in the context of infectious disease such as Mpox. KAPs stand for knowledge, attitudes, and practices taken together [31].

For the majority of diseases for which vaccines currently exist, higher vaccination rates are very important to cover most of the populations or all of them if possible because this generates higher immunity rates. Therefore, in order to create the necessary demand for vaccines, it is not only crucial to develop safe and effective vaccines but also to ensure that the necessary logistical issues, equitable distribution, and the population acceptance are addressed [49]. Then, as part of preventive campaigns, which currently include the Mpox vaccination, vaccine reluctance and acceptance is a critical factor in determining vaccination coverage. This factor should be evaluated and, if necessary, addressed with evidence, education, and promotion. Therefore, this systematic review and meta-analysis aimed to assess various levels of KAPS, awareness, willingness to get vaccinated, worry, and perceptions of risk toward Mpox.

The findings of the present study can be summarized as follows: less than half had good knowledge, while the majority had good attitudes toward Mpox. The majority had willingness to take the Mpox vaccine, and the majority were reported to be aware of Mpox. Less than half of the included participants had worries and perception of risk toward Mpox. Knowledge was highest in the general population, followed by HCWs, and then medical students, while HCWS were the most willing population to be vaccinated against Mpox followed by general population.

Regarding the results of the subgroup analysis of the level of knowledge, the unexpected result can be attributed to the limited number of research undertaken on the knowledge of medical students, with only three studies comprising 722 participants, and all of them confined to Asia. In contrast, research targeting HCWs have a larger sample size and a wider range of study locations, therefore the findings are more credible in this demographic than in medical students. However, because medical students and HCWs have a low percentage of knowledge when compared to the general public, we advocated raising their education level by providing them lectures on Mpox-related topics. Furthermore, future studies on medical students are needed with a bigger sample size and in different continents.

Knowledge about Mpox was measured among different studies and a higher percentage was reported to have poor knowledge (53.4 %). Similar to the current findings, Lounis and Riad [50] in their systematic review showed that even among healthcare professionals and university students, the findings of the various studies' assessments of knowledge about Mpox generally indicated a poor to moderate level, despite using of multiple scales ([34], [51]. Individuals in non-endemic countries may be just discovering Mpox, which could account for such a lack of knowledge [50].

Age, gender, education level, and professional background all appear to influence knowledge about Mpox. Several studies, like those by Sallam et al. (a) [19] and Alshahrani et al. (C) ([33], reported that females were more informed than males, but Sahin et al. [32] found the opposite. This shows that gender may have an impact on knowledge levels, but the direction of that influence may be determined by other factors, such as cultural or societal standards.

In terms of education and professional background, Temsah et al. (a) [24] and Alshahrani et al. (b) [34] revealed that higher education levels and certain professional backgrounds were related with more knowledge. However, Hassan et al. discovered a paucity of knowledge among Bangladeshi physicians, demonstrating that professional experience does not always imply high knowledge levels. Interestingly, Lin et al. [36] discovered that clinical dentistry students had more knowledge than preclinical dental students, although Jairon et al. [41] indicated that undergraduate students knew less. Kumar et al. [23] showed that postgraduates, particularly medical students, have higher levels of knowledge.

Age also appears to affect knowledge levels. Sallam et al. (a) [19] found that older age was related with reduced knowledge, but Sahin et al. [32] observed that knowledge increased with age. These inconsistent results suggest that the relationship between age and knowledge is complicated and impacted by other factors.

Sallam et al. (a) [19] also emphasized the importance of conspiracy theories, discovering that individuals affiliated with non-medical institutions or faculties who believed in conspiracy theories concerning new viral infections had lower levels of expertise. This implies that misinformation can reduce knowledge levels.

Our systematic review and meta-analysis showed that 65 % of the participants are willing to get vaccinated while the rest are not, and this can’t be considered a high percentage since herd immunity requires more than 80 % of population vaccination. Therefore, people must be advised to get the Mpox vaccine to decrease the infection rates. Lower overall percentage was presented by Ulloque-Badaracco et al. [52] in their systematic review and meta-analysis. The goal of their research was to determine the global prevalence of vaccination acceptance for Mpox. Based on the incidence and likely related perception of risk and harm, their findings showed a moderate prevalence of Mpox vaccine acceptance (56 %), which was, as predicted, higher in Europe (70 %) and lower in Asia (50 %).

With regards to the main cause of Mpox spread, Alshrani et al (a) [35] and Sallam et al (b) [20] reported that the most common route of spread was homosexuality among men. Kumar et al. [23] and Wang et al [26] reported that the majority of participants (67 % and 68.88 % respectively) were willing to get vaccinated. On the other hand, Riad et al [25] reported that only 8.8 % accepted to get vaccinated and attributed this to inadequate levels of factual knowledge, particularly about Mpox vaccines and treatments. In addition, Peptan et al [48] reported that only 29.3 % accepted to get vaccinated. Factors associated with not getting vaccinated were various including woman gender [27], older age [24], [26], high-income level [26], and high level of education [24]. Ghazy et al [29] reported that complacency is a psychological determinant of Mpox vaccination, people who are complacent think that vaccination is unneeded because their immune system can defend them. However, Temsah et al (b) [46] reported that perception of Mpox as a dangerous disease, worry about spreading the disease and high commitment to infection precautionary measures were predictors of acceptance of Mpox vaccination. In addition, Wang et al [26] reported that being more concerned about Mpox than COVID-19 and having more information about Mpox were related to a greater desire to consider vaccination.

The majority of participants (71.9 %) were reported to have good attitudes toward Mpox. This means that these people are reacting well toward the Mpox as they put in mind that this is a dangerous infection that may infect them or their families, and friends. They consider it a risk to be an epidemic and they are eager to know the preventive measures and how to avoid getting infected. Many factors are reported to be associated with positive attitudes including male gender, medical students, urban residence, and older age [53], [54], [55].

After the huge pandemic of COVID-19, the appearance of another infectious disease steers panic among the general population as they fear the occurrence of another killing pandemic. The lessons learned from the COVID-19 pandemic emphasize how important it is to fully comprehend every facet of the illness and to take preventative action in case another spike occurs [56]. Therefore, we assessed the levels of worry among the population and compared this to COVID-19. The present systematic review and meta-analysis showed that less than half of the participants were reported to be worried about Mpox (42.7 %). Wang et al [26] reported that 33.2 % of the participants were more worried about Mpox than COVID-19. Temsah et al (b) [46] also reported that worry levels among the general population were higher towards COVID-19 than Mpox during the first month of the WHO announcement. Regarding factors affecting worries, Ajman et al [42] reported that Male HCWs were less worried about Mpox than females, medical students were significantly more worried compared to the other participants, and HCWs who previously developed COVID-19 were significantly less worried about the Mpox outbreak. In contrast, Gallè et al [28] reported that a low level of worry about the transmission of Mpox infection was found.

Regarding awareness towards Mpox, 66 % of participants were reported to be aware. This was in disagreement with the study of Lounis and Riad [50] in the beginning of 2023. The review's conclusions indicated a mediocre degree of Mpox awareness. The absence of awareness about the present outbreak noted among HCWs and medical students is surprising, especially considering that 24.8 % and 27 % of dental professionals [45] and HCWs [38], respectively, never heard about Mpox prior to the outbreak. This is understandable given that the illness usually occurs in endemic countries. These findings may be explained by considering which the studies were carried out in the early stages of the diseases' development and that no cases were documented in the nations where they were carried out [50].

Lin et al [36] reported that preclinical and clinical dental students were aware of the presence of Mpox (89.5 % and 94.4 %, respectively). In addition, Peng et al [37] reported that medical workers over the age of 50 who worked in the infectious diseases, dermatology, and venereal diseases departments and correctly answered the Mpox transmission route were more aware of the Mpox.

Regarding the perception of risk, 40 % of the participants were reported to have a perception of risk towards Mpox. Peng et al [37] reported that approximately 71.8 % of people expressed Mpox perceptions. Winters et al [27] and Temsah et al (b) [46] reported that perception of Mpox risk was correlated with the willingness to get vaccinated.

During a pandemic, handling disaster risks can be very difficult. Authorities must therefore put unique plans and regulations in place to deal with a variety of risks at such times. Disaster management proficiency is directly correlated with the amount of insight acquired from the ongoing experience (COVID-19 pandemic) [57], [58]. Similar to the COVID-19 pandemic, Mpox is a health, political, and socioeconomic crisis that, if left unchecked, could have detrimental effects on society [59]. Positively, during the course of the last four years, important experiences and lessons have been gained in the battle against the COVID-19 pandemic; with their application, it will be simple to prevent and control the virus's reemergence [57]. According to the literature review, there are a number of factors that contribute to Mpox re-emerging, particularly in endemic areas and developing nations. These factors include a lack of information regarding the transmission route and possible reservoir hosts, insufficient training and experience for healthcare professionals, the high cost of detection techniques, and a lack of public health intervention strategies [60], [61], [62]. Therefore, it is recommended to apply public health strategies in fighting infections and preventing their change into pandemics. This includes avoidance of risk factors, mass coverage with vaccinations, early screening and adequate management [63].

Although we included all the available outcomes that can assess the world’s reactions toward Mpox as a potentially emerging pandemic, several limitations exist in our study. This includes inconsistency among the included articles in their sample sizes, their measured outcomes, the differences in the populations, countries, and other demographic data. In addition, cross-sectional studies can’t provide causal relationships. Therefore, further longitudinal studies are recommended to assess knowledge, awareness, attitudes, and willingness to get vaccinated. Also, studies to increase these variables are required in order to be able to combat the virus.

## 5 Conclusion

In the current study, we provide insights into the attitudes and the reaction inquired by the world toward a newly emerging virus soon after COVID-19 which made different mixed reactions encountered by the world’s population. According to the current study, knowledge toward Mpox is required to be increased which can be achieved through awareness campaigns and through the social media. Moreover, people should be advised on the Mpox vaccination to provide herd immunity against this virus as we targeted during the COVID-19 pandemic. This vaccination should be especially increased in vulnerable groups such as chronic disease patients and LGBT communities who are at higher risk for severe diseases. If adequate management and prevention strategies are implemented in the early steps, the virus will be controlled adequately.

## Ethics approval

Not applicable.

## Patient consent

Not applicable.

## Authors’ contribution

All authors made substantial contributions to conception and design, and literature search; took part in drafting the article or revising it critically for important intellectual content; agreed to submit to the current journal; gave final approval of the version to be published; and agreed to be accountable for all aspects of the work.

## CRediT authorship contribution statement

**Mohammad Tanashat:** Writing – review & editing, Writing – original draft, Methodology, Data curation, Conceptualization. **Obieda Altobaishat:** Writing – review & editing, Writing – original draft, Methodology, Data curation. **Abdulrahman Sharaf:** Writing – review & editing, Writing – original draft, Data curation. **Mostafa Hossam El Din Moawad:** Writing – review & editing, Writing – original draft, Data curation. **Mohammad Al-Jafari:** Writing – review & editing, Writing – original draft, Methodology, Data curation, Conceptualization. **Abdulqadir J. Nashwan:** Writing – review & editing, Writing – original draft.

## Declaration of competing interest

The authors declare that they have no known competing financial interests or personal relationships that could have appeared to influence the work reported in this paper.

## Data Availability

Data will be made available on request.
